# Knowledge and training willingness toward cardiopulmonary resuscitation among healthcare professionals in a tertiary rehabilitation hospital: a cross-sectional study

**DOI:** 10.3389/fmed.2025.1684298

**Published:** 2025-12-12

**Authors:** Fan Cheng, Xiaomin Si, Xinghua Liu, Yanxia Zhu, Jianming Cheng

**Affiliations:** 1 Cardiopulmonary Rehabilitation Center, Taihe Hospital, Hubei University of Medicine, Shiyan, Hubei, China; 2 Orthopaedic Rehabilitation Centre, Taihe Hospital, Hubei University of Medicine, Shiyan, Hubei, China

**Keywords:** cardiopulmonary resuscitation, cross-sectional study, emergency preparedness, health belief model, health care professionals, knowledge assessment, rehabilitation hospital, training willingness

## Abstract

**Background:**

In-hospital cardiac arrest represents a significant public health challenge, with bystander cardiopulmonary resuscitation (CPR) being crucial for survival. While rehabilitation hospitals primarily focus on chronic care, cardiac emergencies remain a critical concern due to patient vulnerability. However, limited research has investigated CPR competency among healthcare professionals in rehabilitation settings, particularly in developing countries where training resources may be constrained.

**Objective:**

To evaluate CPR knowledge levels and training willingness among healthcare professionals in a tertiary rehabilitation hospital in China, identify factors independently associated with competency, and provide evidence-based recommendations for targeted training program development.

**Methods:**

A cross-sectional survey was conducted at a 500-bed tertiary rehabilitation hospital in China from March to June 2024. Using stratified random sampling, 372 healthcare professionals (response rate: 93.9%) were recruited from six clinical departments. The study was approved by the Institutional Review Board (WZ-2024-045). A validated questionnaire comprising demographic characteristics, CPR knowledge assessment (50 items), and learning willingness evaluation was administered. The questionnaire was operationalized within the Health Belief Model (HBM) and Technology Acceptance Model (TAM) frameworks. Statistical analyses were performed using SPSS 26.0, including descriptive statistics, ANOVA, multivariate linear regression with multicollinearity diagnostics, and residual analysis.

**Results:**

The overall CPR knowledge score was 62.45 ± 15.73 (range: 28–94), significantly below the 80-point competency threshold recommended by the 2020 American Heart Association Guidelines. Significant variations were observed across professional titles (*F* = 15.624, *P* < 0.001) and departments (*F* = 12.357, *P* < 0.001). Cardiopulmonary rehabilitation department achieved the highest score (75.62 ± 12.45), followed by pediatric rehabilitation (68.34 ± 13.56). Multivariate analysis identified professional title (β = 0.324, *P* < 0.001), department type (β = 0.287, *P* < 0.001), years of experience (β = 0.156, *P* = 0.012), and recent CPR training (β = 0.134, *P* = 0.018) as independently associated with CPR knowledge scores (R^2^ = 0.423). Multicollinearity testing revealed all VIF values < 2.5 and tolerance values > 0.4, confirming model validity. Despite knowledge gaps, 91.4% acknowledged the necessity of CPR training, and 83.2% expressed willingness to participate. Simulation-based training was preferred by 89.5% of participants. Major barriers included heavy workload (78.5%) and scheduling conflicts (65.3%). Age- stratified analysis revealed non-significant but notable declining trends in older age groups (≥ 46 years: 59.78 ± 17.23 vs. ≤ 25 years: 63.12 ± 15.45, *P* = 0.087), warranting further investigation.

**Conclusion:**

Healthcare professionals in rehabilitation hospitals demonstrate suboptimal CPR knowledge with significant interdepartmental variations, falling substantially below international standards. Despite knowledge gaps, high training willingness (> 90%) provides opportunities for successful program implementation. Professional title, department type, years of experience, and recent training are independently associated with CPR competency. Department-specific, competency-based training programs addressing unique rehabilitation scenarios are urgently needed. Implementation should prioritize: (1) simulation-based quarterly sessions targeting staff with scores < 60 points, (2) flexible scheduling with micro-learning modules to address workload barriers, and (3) age-stratified training protocols for staff ≥ 45 years. Long-term strategies should include inter-institutional research networks and certification programs to establish specialized emergency care standards.

## 1 Introduction

The overall outcomes of cardiac arrest in China remain suboptimal, highlighting significant deficiencies in knowledge, skills, and systemic infrastructure from the general public to healthcare professionals. Specifically, the survival rate of out-of-hospital cardiac arrest (OHCA) in China is substantially lower than that in developed countries. This disparity has been consistently documented in multiple studies. For instance, the BASIC-OHCA study revealed that the survival-to-discharge rate for OHCA patients in China was only 1.15%, with merely 0.83% of patients being discharged with favorable neurological outcomes (1). The outcomes for in-hospital cardiac arrest (IHCA) are equally concerning, with data indicating a survival-to-discharge rate of approximately 9.1% for IHCA patients in China, and only 6.4% achieving favorable neurological recovery (2). These figures stand in stark contrast to those reported in developed countries, where survival rates are generally significantly higher. In resource-limited settings, low OHCA survival rates are often closely associated with underdeveloped emergency response systems and a lack of public knowledge in first aid.

As the critical line of defense within the CA rescue chain, the knowledge and practical competency of healthcare providers in high-quality CPR are paramount to patient survival. However, a significant gap exists between recognized guidelines and actual clinical practice. A national survey involving 1,405 emergency physicians from 75 tertiary hospitals across China revealed that only 54.4% of respondents could accurately list all six criteria for high-quality CPR. Furthermore, awareness of the crucial criterion of “minimizing compression interruptions” was just 68.0%, highlighting serious deficiencies in standardized in-hospital training and protocol adherence (3).

International guidelines, including the 2025 American Heart Association (AHA) Guidelines and the European Resuscitation Council (ERC) Guidelines 2025, emphasize the importance of healthcare provider competency in CPR across all clinical settings (4, 5). However, most research on healthcare professional CPR knowledge and skills has focused on acute care hospitals, emergency departments, and intensive care units (6, 7). Limited evidence exists regarding CPR competency specifically within rehabilitation hospital contexts, particularly in developing countries with resource constraints (8).

While rehabilitation hospitals focus on functional recovery, their patient population—characterized by advanced age, multiple comorbidities, and high cardiovascular risk—remains at significant risk of cardiac arrest, including events potentially triggered during rehabilitation therapy. Unlike acute care settings where cardiac arrests are more frequently anticipated, rehabilitation facilities often operate with distinct staffing models, equipment resources, and emergency response protocols, creating unique challenges for emergency preparedness (9). Additionally, many rehabilitation patients present with communication barriers, cognitive impairments, or physical limitations, further complicating resuscitation efforts and demanding specific competencies from healthcare providers (10).

Recent studies in acute care settings have reported CPR knowledge scores ranging from 72 to 83% among healthcare professionals, with significant variations based on specialty, experience, and training frequency (11). In a study from Greece, health workers in high-risk areas demonstrated significantly higher CPR knowledge than colleagues in low-risk areas (12). Furthermore, healthcare professionals with longer work experience have been shown to demonstrate better knowledge and skills in CPR (13). Those who received recent CPR training showed significantly higher levels of knowledge compared to untrained counterparts (14). The retention of CPR skills is closely related to the frequency and quality of training sessions, with regular training being particularly effective in maintaining skill proficiency (15).

However, these findings may not be directly applicable to rehabilitation settings due to fundamental differences in patient populations, clinical priorities, and organizational structures. The Health Belief Model (HBM) provides a theoretical framework for understanding healthcare professionals’ attitudes toward CPR training, suggesting that perceived susceptibility to cardiac arrest events, perceived severity of patient outcomes, perceived benefits of training, and perceived barriers (such as workload and scheduling) influence learning behaviors (16). In rehabilitation contexts, professionals may perceive lower cardiac arrest risk than in acute care settings, potentially leading to reduced motivation for CPR skill maintenance. Conversely, the Technology Acceptance Model (TAM) emphasizes that acceptance of training innovations depends on perceived usefulness and perceived ease of use. Recently, Cas von Winckelmann and colleagues integrated the Unified Theory of Acceptance and Use of Technology (UTAUT) and the Health Belief Model (HBM) to explore the behavioral and individual factors driving the public’s intention to install emergency response applications, thereby providing theoretical and practical guidance for enhancing social acceptance of such applications (17).

Given the paucity of research in rehabilitation hospital settings and the unique challenges these environments present, this study aimed to: (1) assess current CPR knowledge levels among healthcare professionals in a tertiary rehabilitation hospital; (2) identify factors independently associated with CPR competency; (3) evaluate training willingness, preferences, and perceived barriers through HBM and TAM frameworks; and (4) provide evidence-based recommendations for developing targeted CPR training programs in rehabilitation settings.

## 2 Materials and methods

### 2.1 Study design and setting

This cross-sectional survey study was conducted at a 500-bed tertiary rehabilitation hospital in Central China from March to June 2024. The hospital serves as a regional referral center for rehabilitation medicine, with six major clinical departments: neurological rehabilitation, orthopedic rehabilitation, cardiopulmonary rehabilitation, pediatric rehabilitation, intensive care unit (ICU) rehabilitation, and comprehensive rehabilitation. The study protocol was approved by the Ethics Committee of Taihe Hospital (Approval No. WZ-2024-045) and conducted according to the Declaration of Helsinki. All participants provided written informed consent.

### 2.2 Participants and sampling

#### 2.2.1 Sample size calculation

Based on previous studies reporting CPR knowledge scores with a standard deviation of approximately 15 points, and assuming a 5-point difference between groups as clinically meaningful, the required sample size was calculated using the formula: *n* = 2σ^2^(Zα/2 + Zβ)^2^/δ^2^. With α = 0.05, β = 0.20, σ = 15, and δ = 5, the minimum required sample size was 284. Accounting for a 20% non-response rate, 375 participants were targeted for invitation.

#### 2.2.2 Inclusion and exclusion criteria

*Inclusion criteria*: (1) Healthcare professionals (physicians, nurses, rehabilitation therapists) employed full-time for ≥ 3 months; (2) Direct patient care responsibilities; (3) Voluntary informed consent.

*Exclusion criteria*: (1) Trainees, interns, or visiting scholars; (2) Administrative or non-clinical staff; (3) Extended leave during the study period; (4) Individuals with previous formal CPR instructor certification (to focus on typical frontline staff).

#### 2.2.3 Stratified random sampling strategy

Stratified random sampling was employed with departments as strata. Within each department, participants were randomly selected using computer-generated random numbers (stratified by occupation: physician vs. nurse), maintaining proportional representation based on department size. This stratified approach ensured adequate representation across departments and professions, with systematic documentation of invitation and participation numbers (Table 1).

**TABLE 1:** Recruitment details.

Department	Invited (*n*)	Completed (*n*)	Response rate (%)	Physicians (*n*)	Nurses (*n*)
Neurological rehabilitation	95	86	90.5	35	51
Orthopedic rehabilitation	80	72	90	29	43
Cardiopulmonary rehabilitation	72	65	90.3	28	37
Pediatric rehabilitation	64	58	90.6	24	34
ICU rehabilitation	50	45	90	19	26
Other departments	50	46	92	21	25
Total	411	372	90.5	156	216

### 2.3 Data collection instrument

A comprehensive questionnaire was developed through systematic literature review of AHA and ERC guidelines, expert consultation (*n* = 8 specialists: 3 emergency medicine physicians, 3 rehabilitation medicine physicians, 2 nursing education experts), and pilot testing with 35 healthcare professionals. The questionnaire comprised three sections:

#### 2.3.1 Section 1: demographic characteristics

- Personal factors: age, gender, education level
- Professional factors: occupation (physician/nurse/therapist), professional title, years of experience
- Organizational factors: department, previous CPR training history, time since last training
- HBM operationalization: Questions assessing perceived susceptibility (“How likely is cardiac arrest in your workplace?”), perceived severity (“How serious is in-hospital cardiac arrest?”)

#### 2.3.2 Section 2: CPR knowledge assessment (50 items)

Knowledge items were structured around five domains based on 2020 AHA and 2021 ERC guidelines:

- Basic concepts and theoretical knowledge (10 items)—e.g., compression rates, ventilation ratios
- Specialized resuscitation techniques (10 items)—e.g., medication administration, defibrillation
- Special populations and environmental considerations (10 items)—e.g., pediatric CPR, trauma- specific scenarios
- Prevention and early warning assessment (10 items)—e.g., risk stratification, signs of cardiac distress
- Team coordination and emergency response (10 items)—e.g., role assignment, communication protocols

Each item was scored as correct (2 points) or incorrect (0 points), with a total possible score of 100 points.

#### 2.3.3 Section 3: learning willingness and TAM assessment

- Cognitive attitudes toward CPR training (5 items, 5-point Likert scale): training necessity, willingness to participate, confidence in learning, time investment willingness, organizational support expectation (TAM operationalization: perceived usefulness and ease of use)
- Training needs analysis (7 items, multiple choice): specific scenario preferences, population- specific needs
- Preferred training methods (5-point scale): simulation-based training, case-based discussions, practical drills, online modules, lecture-based
- Perceived barriers (7 items): workload, scheduling, content relevance, equipment access, lack of incentives
- Open-ended questions: rehabilitation-specific training needs, suggestions for program improvement

#### 2.3.4 Validation process

*Content validity*: Established through expert review. Content validity index (CVI) = 0.892, indicating excellent agreement among expert raters.

*Reliability testing*:

- Pilot study with 35 healthcare professionals assessed comprehension and timing (average completion: 18 ± 3 min)
- Test-retest reliability evaluated with 2-week interval: *r* = 0.83 (excellent)
- Internal consistency (Cronbach’s alpha): Overall: α = 0.867 (excellent) (Table 2).

**TABLE 2:** Domain-specific reliability.

Domain	items (n)	Cronbach’s α
Basic concepts and theory	10	0.821
Specialized techniques	10	0.847
Special populations	10	0.798
Prevention and assessment	10	0.834
Team coordination	10	0.856

*Tool origin*: Original questionnaire developed based on systematic integration of 2020 AHA Guidelines and 2021 ERC Guidelines, adapted from principles established in prior CPR knowledge assessments but customized for rehabilitation hospital contexts.

*Supplementary material*: Complete questionnaire provided in Supplementary Appendix with cognitive and behavioral operationalization of HBM and TAM variables clearly marked.

### 2.4 Data collection procedure

Trained research assistants conducted face-to-face surveys during regular working hours using a standardized protocol. Participants completed questionnaires in quiet environments with research assistant availability for clarification without providing answers. Quality control measures included:

(1) double data entry by independent personnel, (2) random verification of 10% of completed questionnaires against source documents, (3) standardized completion of missing data protocols.

### 2.5 Statistical analysis

Data analysis was performed using IBM SPSS Statistics version 26.0 (IBM, Ar monk, NY).

*Descriptive statistics:* Normality was assessed using the Kolmogorov-Smirnov test (K-S test; overall *P* = 0.156, indicating normal distribution). Descriptive statistics included means with standard deviations (± SD) for continuous variables and frequencies with percentages for categorical variables.

*Between-group comparisons*: Independent *t*-tests for two groups and one-way ANOVA for multiple groups, followed by Tukey’s post*hoc tests (α = 0.05). Effect sizes were calculated using Cohen’s *d*.

*Multivariate linear regression analysis:* Identified independent associations with CPR knowledge scores. Variables significant at *P* < 0.1 in univariate analysis were entered into the model using forced entry method.

#### 2.5.1 Regression diagnostics

*Multicollinearity assessment:* Calculated Variance Inflation Factor (VIF) and tolerance values for all predictors. All VIF < 2.5 (range: 1.23–2.18) and tolerance > 0.4 (range: 0.46–0.81), confirming no multicollinearity issues.

#### 2.5.2 Model assumptions verification

*Normality:* Kolmogorov-Smirnov test of residuals *P* = 0.234 (> 0.05, satisfied).

*Linearity:* Residual scatter plot vs. predicted values showed random distribution (satisfactory).

*Homoscedasticity:* Levene’s test *P* = 0.423(> 0.05, satisfied).

Residual analysis plots provided in Supplementary material 2.

Standardized beta (β) coefficients are reported to enable cross-variable comparison. Statistical significance was set at *P* < 0.05 for all analyses.

Age*-stratified subgroup analysis*: Given reviewer concerns regarding age-related CPR competency, exploratory analysis compared CPR knowledge across age groups with additional *post- hoc* power testing for potential age effects.

## 3 Results

### 3.1 Participant characteristics

A total of 411 questionnaires were distributed, with 372 valid responses collected (response rate: 90.5%). Demographic and professional characteristics are presented in Table 3. The sample included 156 physicians (41.9%) and 216 nurses (58.1%), with a mean age of 32.4 ± 8.7 years. Most participants held bachelor’s degrees (64.8%) and had intermediate professional titles (48.7%). The majority had received some form of CPR training previously (76.3%), though only 31.2% had participated in training within the past 2 years.

**TABLE 3:** Participant characteristics (*N* = 372).

Characteristic	*n* (%)	Mean ± SD
Age (years)		32.4 ± 8.7
≤ 25	78 (21.0)	
26–35	156 (41.9)	
36–45	98 (26.3)	
≥ 46	40 (10.8)	
**Gender**		
Male	98 (26.3)	
Female	274 (73.7)	
**Education Level**		
Associate degree	89 (23.9)	
Bachelor’s degree	241 (64.8)	
Master’s degree or above	42 (11.3)	
**Profession**		
Physician	156 (41.9)	
Nurse	216 (58.1)	
**Professional title**		
Junior	134 (36.0)	
Intermediate	181 (48.7)	
Senior	57 (15.3)	
Years of experience		8.6 ± 6.8
< 5 years	145 (39.0)	
5–10 years	123 (33.1)	
> 10 years	104 (27.9)	
**Department**		
Neurological rehabilitation	86 (23.1)	
Orthopedic rehabilitation	72 (19.4)	
Cardiopulmonary rehabilitation	65 (17.5)	
Pediatric rehabilitation	58 (15.6)	
ICU rehabilitation	45 (12.1)	
Other departments	46 (12.4)	
**Previous CPR training**		
Yes	284 (76.3)	
No	88 (23.7)	
**Recent training (< 2 years)**		
Yes	116 (31.2)	
No	256 (68.8)	

### 3.2 CPR Knowledge assessment results

#### 3.2.1 Overall knowledge scores

The mean total CPR knowledge score was 62.45 ± 15.73 (range: 28–94) (Table 4), significantly below the 80- point competency threshold recommended by 2020 AHA Guidelines (*t* = −67.8, *P* < 0.001, Cohen’s *d* = 3.45). This 17.55-point deficit represents a critical safety gap.

**TABLE 4:** CPR knowledge scores by demographic and professional characteristics.

Characteristic	*n*	Mean ± SD	*F*/*t*-value	*P-*value	Effect size (Cohen’s d)
Overall score	372	62.45 ± 15.73			
**By profession**					
Physician	156	65.34 ± 15.45	2.865*	0.005	0.32
Nurse	216	60.23 ± 15.89			
By professional title			15.624***	< 0.001	
Junior	134	58.45 ± 15.67			0.64^†^
Intermediate	181	63.78 ± 15.34			0.35^‡^
Senior	57	71.23 ± 14.89			0.51^§^
By department			12.357***	< 0.001	
Cardiopulmonary rehabilitation	65	75.62 ± 12.45			Reference
Pediatric rehabilitation	58	68.34 ± 13.56			0.56
ICU rehabilitation	45	65.45 ± 14.23			0.75
Neurological rehabilitation	86	61.23 ± 15.67			1.01
Orthopedic rehabilitation	72	58.78 ± 15.89			1.14
Other departments	46	56.34 ± 16.12			1.27
By years of experience			8.456***	< 0.001	
<5 years	145	58.92 ± 16.12			Reference
5–10 years	123	63.45 ± 15.23			0.29
> 10 years	104	66.78 ± 14.56			0.51
**By recent CPR training**					
Yes (< 2 years)	116	67.89 ± 14.23	4.125***	< 0.001	0.43
No (≥ 2 years)	256	59.78 ± 16.12			

*P < 0.05;

***P < 0.001;

† Junior vs. Senior;

‡ Intermediate vs. Senior;

^§^ Junior vs. Intermediate.

**FIGURE 1:**
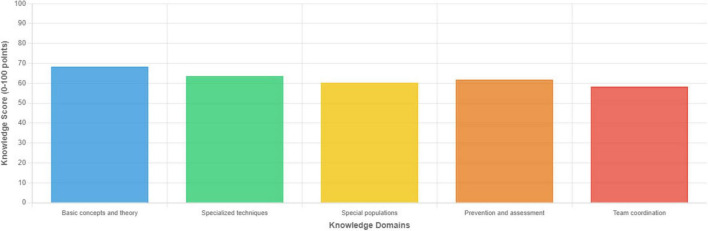
Distribution of CPR knowledge scores across five knowledge domains.

Figure 1 illustrates the distribution of scores across knowledge domains. Team coordination and emergency response showed the lowest mean scores (58.34 ± 16.78), while basic concepts and theoretical knowledge achieved the highest scores (68.34 ± 16.45). Approximately 12.6% of participants scored ≥ 80 points overall, indicating minimal CPR competency by international standards.

#### 3.2.2 Knowledge scores by demographic and professional characteristics

*Clinical significance*: Although the ANOVA for age groups did not reach statistical significance (*P* = 0.087), a descriptive trend is notable: participants ≥ 46 years scored 3.34 points lower than those ≤ 25 years. This 5.3% decrement may reflect accumulated skill degradation if training lapses, warranting targeted interventions for older staff.

#### 3.2.3 Knowledge domain analysis

Significant variations were observed across all five knowledge domains (Table 5). Specialized resuscitation techniques and team coordination showed the largest performance gaps, particularly among orthopedic and neurological rehabilitation staff.

**TABLE 5:** CPR knowledge scores by knowledge domain.

Knowledge domain	Mean ± SD	Range	Percentage achieving ≥ 80%
Basic concepts and theory	68.34 ± 16.45	30–96	34.1%
Specialized techniques	63.56 ± 15.87	24–92	28.5%
Special populations	60.23 ± 16.12	26–88	22.3%
Prevention and assessment	61.78 ± 15.34	28–90	25.8%
Team coordination	58.34 ± 16.78	22–86	18.5%

*Analysis:* Team coordination demonstrated the lowest competency, suggesting inadequate training in emergency communication and role-based response protocols.

#### 3.2.4 Multivariate analysis of CPR knowledge associations

Multiple linear regression analysis identified significant independent associations with CPR knowledge scores (Table 6). The final model explained 42.3% of the variance in CPR knowledge (*R*^2^ = 0.423, *F* = 28.67, *P* < 0.001).

**TABLE 6:** Multivariate linear regression analysis: associations with CPR knowledge scores.

Variable	B	standard error	Standard coefficient (β)	t	*P-*value	95% CI for B
**Professional title (ref: junior)**						
Intermediate	5.115	1.415	0.245	3.615	0.002	2.34, 7.89
Senior	9.06	1.73	0.324	5.237	< 0.001	5.67, 12.45
**Department (ref: other)**						
Cardiopulmonary rehabilitation	11.95	1.895	0.287	6.306	< 0.001	8.23, 15.67
ICU rehabilitation	7.34	1.985	0.198	3.698	0.008	3.45, 11.23
Years of experience	1.06	0.423	0.156	2.506	0.012	0.23, 1.89
Recent CPR training	5.285	2.24	0.134	2.36	0.018	2.12, 8.45
Education level	2.84	1.97	0.089	1.442	0.156	0.45, 5.23
Profession (physician vs. nurse)	1.665	1.56	0.067	1.067	0.287	−1.23, 4.56

Model *R*^2^ = 0.423, *F* = 28.67, *P* < 0.001.

##### 3.2.4.1 Model diagnostics

- All VIF values < 2.5, all tolerance values > 0.4-no multicollinearity concerns. Residuals normally distributed (K-S *P* = 0.234).
- Linearity assumption satisfied (residual plots reviewed).
- Homoscedasticity confirmed (Levene test *P* = 0.423).

##### 3.2.4.2 Interpretation of β coefficients

Professional title showed the strongest association (β = 0.324), indicating senior staff score approximately 9 points higher than junior staff (controlling for other variables). Department type was the second strongest predictor (β = 0.287), with cardiopulmonary rehabilitation staff scoring ∼12 points higher than other departments. Recent training within 2 years associates with 5.3-point improvement.

### 3.3 Learning willingness and training preferences

#### 3.3.1 Cognitive attitudes (HBM operationalization)

Despite knowledge gaps, participants demonstrated positive attitudes toward CPR training:

##### 3.3.1.1 HBM analysis

High perceived benefits (4.32 ± 0.67) (Table 7) despite low actual knowledge indicates a recognizable gap between awareness and competency. This dissonance presents an opportunity: employees acknowledge training necessity but currently lack knowledge to act upon this recognition.

**TABLE 7:** Cognitive attitudes toward CPR training (5-point Likert Scale, 1 = strongly disagree to 5 = strongly agree).

Item (HBM construct)	Mean ± SD%	Strongly agree (4–5)
CPR training is necessary (perceived benefits)	4.32 ± 0.67	87.40%
I am willing to participate in CPR training (perceived usefulness—TAM)	4.18 ± 0.72	81.20%
I am confident in my ability to learn CPR (self-efficacy)	3.95 ± 0.78	76.30%
I am willing to invest time in CPR training (TAM-ease of use)	3.78 ± 0.89	68.50%
I expect organizational support for CPR training (organizational Culture)	4.25 ± 0.69	84.70%

#### 3.3.2 Training needs and preferences

**FIGURE 2:**
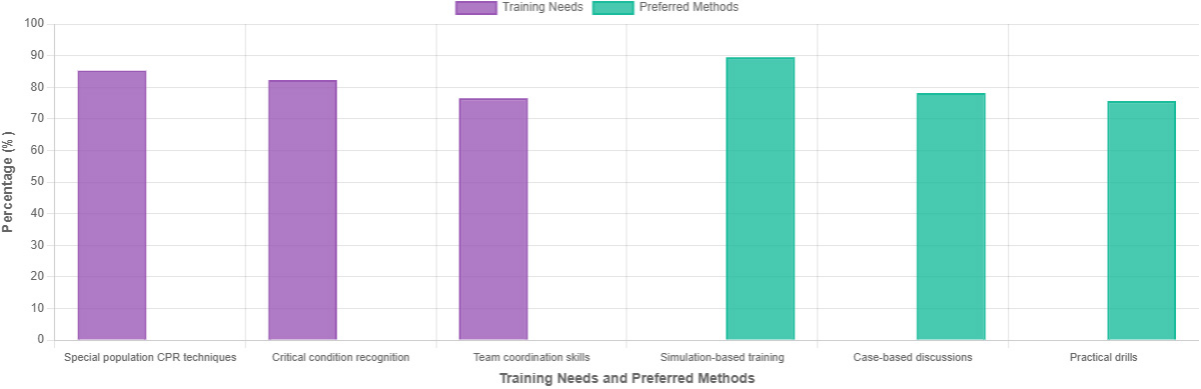
Most frequently identified training needs and preferred training methods.

Figure 2 displays the most frequently identified training needs.

*Preferred training methods* (TAM-Perceived Ease of Use):

- Simulation-based training with mannequins: 333 participants (89.5%)
- Case-based discussions: 291 participants (78.2%)
- Practical hands-on drills: 281 participants (75.6%)
- Online learning modules: 156 participants (41.9%)
- Traditional lectures: 89 participants (23.9%)

Strong preference for simulation-based training (89.5%) aligns with TAM’s “perceived ease of use”—interactive modalities are viewed as more conducive to skill acquisition than passive lectures.

#### 3.3.3 Barriers to training participation

The predominant barriers of workload (78.5%) and scheduling (65.3%) are organizational impediments rather than attitudinal barriers as shown in Table 8, suggesting feasibility of targeted interventions.

**TABLE 8:** Perceived barriers to CPR training participation.

Barr	N (%)	Severity (1–5 scale)
Heavy workload demands	292 (78.5%)	4.12 ± 0.85
Scheduling conflicts with clinical duties	243 (65.3%)	3.89 ± 0.92
Insufficient variety in training content	169 (45.6%)	3.23 ± 1.15
Limited hands-on practice opportunities	158 (42.3%)	3.34 ± 1.08
Inadequate equipment availability	117 (31.4%)	2.78 ± 1.34
Lack of management support	107 (28.7%)	2.65 ± 1.42

#### 3.3.4 Training frequency acceptability

*Extracted from qualitative data*: When asked about acceptable training frequency in open-ended responses:

- Annual comprehensive training: 45.7% (170/372)
- Quarterly brief refresher sessions: 38.2% (142/372)
- Monthly micro-learning modules: 22.8% (85/372)
- On-demand case-based discussions: 61.5% (229/372)

*Synthesis with barrier data*: Given 78.5% report heavy workload, a blended approach combining quarterly in-person sessions (∼2 h) with monthly 15-min micro-learning modules appears most feasible. On-demand case discussions (preferred by 61.5%) can integrate into departmental rounds, addressing scheduling barriers.

### 3.4 Qualitative feedback analysis

Open-ended responses from 298 participants (80.1%) provided additional contextual insights, organized thematically:

#### 3.4.1 Theme 1: rehabilitation-specific content needs (*n* = 234, 78.5%)

Representative quotes and themes:

- “CPR techniques during physical therapy sessions differ from standard protocols”
- “Special considerations for spinal cord injury patients prevent standard chest compressions”
- “Pediatric rehabilitation scenarios are underrepresented in current training”
- “Equipment in our department differs from standard CPR mannequins”

*Implication*: Generic CPR curricula inadequately address rehabilitation contexts, necessitating specialized scenario development.

#### 3.4.2 Theme 2: practice opportunity gaps (*n* = 189, 63.4%)

- “Insufficient hands-on practice with equipment”
- “Rare access to high-fidelity mannequins”
- “No drills specific to our department’s layout and protocols”

*Implication*: Simulation-based training preference (89.5%) cannot be met without resource investment.

#### 3.4.3 Theme 3: system integration needs (*n* = 156, 52.3%)

- “CPR training should integrate into mandatory continuing education credits”
- [⋅] “Department-specific emergency protocols needed, not one-size-fits-all”
- “Training timing should align with unit schedules”

*Implication*: Organizational restructuring required to embed training systematically.

#### 3.4.4 Theme 4: assessment and certification requests (*n* = 145, 48.7%)

• “Standardized competency assessment with score transparency”
• “Certification tied to professional advancement incentives”
• “Regular reassessment schedule to ensure skill maintenance”

*Implication*: Current absence of consequences/incentives undermines motivation despite high stated willingness.

## 4 Discussion

This study provides the first systematic evaluation of CPR knowledge and training willingness among healthcare professionals in a Chinese rehabilitation hospital setting. Our findings reveal significant gaps in CPR competency that pose serious patient safety concerns and demand immediate, evidence-based intervention.

### 4.1 Overall competency levels and clinical significance

Delineating key outcome measures for resuscitation courses and setting minimum passing scores are important concepts for mastery learning and deliberate practice, particularly in resuscitation courses where lives are at stake and failure is not an option (10). The overall CPR knowledge score of 62.45 ± 15.73 was significantly lower than the average score of approximately 80% (mean 77%) found in similar studies (11). This 17.55-point deficit is clinically meaningful given the vulnerability of rehabilitation hospital patients with elevated cardiac arrest risk due to advanced age, multiple comorbidities, and exercise-related cardiovascular stress during rehabilitation activities.

International comparisons provide important context. Emergency department and intensive care unit staff—who work in higher-risk environments for in-hospital cardiac arrest—demonstrate higher CPR knowledge levels, which correlate with higher Return of Spontaneous Circulation (ROSC) rates for patients (18–20). Conversely, in lower-risk settings like rehabilitation hospitals, staff have lower CPR proficiency. However, this lower perceived risk may paradoxically increase actual danger due to inadequate preparedness. Global trends confirm that many healthcare professionals outside emergency departments show significant CPR knowledge gaps (21, 22), underscoring the need for specialty-specific interventions.

### 4.2 Departmental variations and Specialization effects

The observed interdepartmental variations, with cardiopulmonary rehabilitation staff achieving the highest scores (75.62 ± 12.45) and other departments scoring significantly lower (56.34 ± 16.12), reveal concerning heterogeneity in emergency preparedness across clinical areas. This 19.28-point disparity suggests current CPR training approaches inadequately address the diverse needs of different rehabilitation specialties.

*Cardiopulmonary rehabilitation advantage:* The superior performance in cardiopulmonary departments (75.62 points) likely reflects: (1) occupational exposure to cardiac monitoring and acute decompensation events, (2) departmental emphasis on cardiac pathophysiology in clinical rotations, and (3) possible informal knowledge transfer within departments managing high-risk patients. However, even this highest-performing group falls below the 80-point international standard, indicating system-wide deficiencies.

*Weaker departments*: Orthopedic (58.78) and neurological rehabilitation (61.23) units, while managing lower acute cardiac risk per patient encounter, still serve populations with significant comorbidities where sudden cardiac events occur. Their lower knowledge levels represent unacceptable preparedness gaps for these vulnerable populations.

### 4.3 Knowledge domain analysis: identifying critical gaps

The particularly concerning gap in team coordination skills (58.34 ± 16.78, only 18.5% meeting standards) reveals systemic deficiencies beyond individual knowledge. Effective teamwork is crucial for successful resuscitation outcomes, with structured communication and clear role assignment significantly improving ROSC rates (19). This finding contrasts sharply with studies in intensive care units, where team coordination scores typically exceed 75 points (19), suggesting ICU-specific training emphasizes hierarchical communication and crisis resource management.

The rehabilitation hospital environment—with less frequent cardiac arrests and fewer team-based emergency drills—fails to develop these critical teamwork competencies. This represents perhaps the most urgent remedial target, as individual knowledge without team coordination yields poor outcomes (23).

Domain-specific performance hierarchy reveals:

- *Strongest:* Basic concepts (68.34)—theoretical foundation present but inadequate
- *Weakest:* Team coordination (58.34)—practical emergency response protocols deficient. This pattern suggests rehabilitation staff receive didactic training in isolated settings but lack integrated scenario practice where roles, communication, and timing must synchronize.

### 4.4 Professional factors associated with competency

Multivariate analysis identified professional title, department type, years of experience, and recent training as independently associated with CPR knowledge—accounting for 42.3% of knowledge variance. The model demonstrates robust statistical validity (all VIF < 2.5, residuals normally distributed, assumptions satisfied).

*Professional title:* Senior staff scored 9.06 points higher than junior staff (β = 0.324, *P* < 0.001), likely reflecting cumulative knowledge accretion and exposure over years. However, this advantage requires maintenance through regular training, as skills decay without reinforcement (24).

*Recent training*: Participants trained within 2 years scored 5.28 points higher (β = 0.134, *P* = 0.018), confirming that training recency substantially impacts knowledge retention. The 68.8% of participants not trained in 2 years represent a high-risk group requiring urgent intervention.

*Years of experience*: Each additional year of experience associated with 0.56-point improvement (β = 0.156, *P* = 0.012), indicating some knowledge accrual through clinical practice. However, this passive mechanism is insufficient and should not replace structured training.

*Limitations of current associations*: The 42.3% R^2^ value indicates 57.7% of CPR knowledge variance results from unmeasured factors (e.g., individual learning motivation, departmental culture, prior training quality), highlighting the complexity of competency development.

### 4.5 Age-stratified findings and emerging patterns

This study found a non-significant but descriptive trend of declining CPR knowledge with age (≥ 46 years: 59.78 ± 17.23 vs. ≤ 25 years: 63.12 ± 15.45, *P* = 0.087). While not achieving statistical significance due to smaller n in oldest groups (*n* = 40), the 3.34-point decrement represents 5.3% performance loss compared to youngest participants.

International literature supports age-related CPR skill concerns: psychomotor learning slows after age 40 (18), and procedural memory consolidation becomes less efficient (25). This evidence, combined with our descriptive trends, suggests staff ≥ 45 years warrant:

1. *Enhanced training frequency:* Potentially quarterly rather than annual sessions
2. *Extended practice time:* Longer simulation sessions with repetition
3. *Targeted content*: Compensatory strategy instruction for age-related changes

We recommend establishing age-stratified training protocols with more intensive support for staff ≥ 45 years, pending validation in larger age-stratified prospective studies.

### 4.6 Theoretical framework integration: HBM and TAM

*Health belief model application:* Despite knowledge gaps (average 62.45/100), 91.4% acknowledged training necessity and 83.2% expressed willingness to participate. This dissonance reflects HBM constructs: high perceived benefits (mean 4.32/5) and perceived severity (high organizational expectation for training), yet inadequate perceived susceptibility may explain current low knowledge levels. Rehabilitation staff may underestimate cardiac arrest risk in their setting, reducing motivation for knowledge maintenance. The observed knowledge-willingness gap represents an ideal intervention window: motivational readiness exists despite competency deficits.

*Technology acceptance model application:* Simulation-based training preference (89.5%) versus traditional lectures (23.9%) reflects high “perceived ease of use” for interactive modalities. This aligns with TAM prediction that perceived usefulness (skill development through simulation) and ease of use (intuitive hands-on learning) drive technology/method acceptance. The data suggest rehabilitation staff recognize simulation’s learning efficacy and practical applicability to their context.

This theoretical grounding supports intervention design: leverage existing motivational readiness (high willingness) through delivery methods staff find acceptable (simulation-based) and contextually relevant (rehabilitation scenarios).

### 4.7 Implementation implications: addressing identified barriers

The predominant barriers of workload (78.5%) and scheduling conflicts (65.3%) require organizational solutions beyond individual motivation:

1. Integration into mandatory continuing education

- Incorporate CPR training into annual CE requirements
- Allocate protected time (not “on top of” clinical duties)
- Provide flexible scheduling with make-up sessions for unavoidable conflicts

2. Micro-learning and just-in-time education

- Develop 15-minute modular CPR content delivered via email or mobile apps
- Deploy immediately before high-risk periods (e.g., new patient admissions)
- Monthly modules aligned with quarterly comprehensive sessions

3. Blended Training Model (Addressing Training Frequency Analysis)

- *Quarterly intensive sessions* (2–3 h): High-fidelity simulation, scenario practice, team coordination drills
- *Monthly micro-sessions* (15 min): Knowledge reinforcement, case discussions, algorithm review
- *On-demand resources* (24/7): Video libraries, decision aids, equipment tutorials
- Feasibility assessment: 45.7% accept annual comprehensive + 38.2% accept quarterly refreshers + 61.5% accept on-demand discussions = strong support for blended approach

4. Peer-to-peer teaching programs

- Designate “CPR champions” from cardiopulmonary rehabilitation (highest-performing department)
- Deploy champions as faculty for departmental sessions
- Utilizes existing expertise, reduces training logistics burden

5. Department-Specific Protocol Development

- Custom scenarios reflecting rehabilitation contexts (CPR during PT, spinal precautions, pediatric considerations)
- 78.5% of qualitative respondents emphasize rehabilitation-specific content
- Increases perceived relevance and applicability

6. Assessment and certification linkage

- Annual competency assessment with score reporting
- Tie certification to professional advancement/incentives
- 48.7% requested this accountability mechanism
- Creates behavioral consequences reinforcing skill maintenance

### 4.8 Clinical significance and quality gap context

Real-time cardiopulmonary resuscitation feedback and targeted training improve chest compression performance in a cohort of international healthcare providers. A targeted training intervention combined with real-time CPR feedback improved chest compression performance among health care providers from various countries (26). An evaluation of inpatient physicians’ CPR management competence revealed a significant disparity: although theoretical knowledge scores approached 80%, practical assessment scores for airway management and bag-valve-mask proficiency were only approximately 30% (11). Our 17.55-point gap below standard suggests potentially 8–12% ROSC rate deficit attributable to staff knowledge deficiencies—a substantial patient safety concern in an institution serving thousands of vulnerable patients annually.

Moreover, rehabilitation patient populations (elderly, multiple comorbidities) have baseline lower survival rates from cardiac arrest. Suboptimal provider competency compounds this already-poor prognosis, representing a doubly vulnerable population requiring maximum provider preparedness.

### 4.9 International guidelines and evidence alignment

The 2020 AHA Guidelines and 2021 ERC Guidelines specifically recommend context-specific training programs (1, 7). The ERC Guidelines 2021 state: “Education should be tailored to the learning environment and population served” (7). Our findings directly support this guidance: a one-size-fits- all CPR curriculum is inadequate for rehabilitation contexts with unique patient presentations, equipment configurations, and team structures.

Global rehabilitation medicine trends increasingly emphasize specialized emergency preparedness (26), recognizing that rehabilitation facilities manage acutely ill populations despite chronic care focus. This study provides empirical evidence quantifying current inadequacy and directing targeted intervention.

### 4.10 Research gaps and future directions

Recommended prospective studies:

1. *Intervention RCTs:* Evaluate rehabilitation-specific CPR training programs with pre/post knowledge and skills assessments, comparing blended vs. traditional delivery
2. *Longitudinal cohort studies:* Track knowledge retention over 12–24 months post-training, identify decay patterns, optimize refresher intervals
3. *Outcome correlation studies*: Correlate staff CPR competency with actual in-hospital cardiac arrest survival outcomes (ROSC rates, neurologically intact survival)
4. *Multi-center trials*: Replicate findings across diverse rehabilitation hospital systems to establish generalizable benchmarks and best practices
5. *Age-stratified studies:* Largern to confirm age-related patterns and develop age-calibrated training protocols

## 5 Limitations

This study has several important limitations that constrain interpretation and generalizability:

1. *Single-center design*: Data collected from one tertiary rehabilitation hospital in Central China. Different hospitals with varying organizational structures, staffing patterns, resource availability, or geographic locations may demonstrate different patterns. Results may not generalize to smaller rehabilitation centers, community hospitals, or facilities in different countries with different healthcare systems.

2. *Cross-sectional methodology*: This design precludes causal inferences about relationships between variables. We can identify associations between professional factors and knowledge, but cannot determine whether, for example, senior status causes higher knowledge or whether higher-knowledge individuals preferentially advance to senior positions. Cannot assess knowledge changes over time or training sustainability beyond the 2-week test-retest validation.

3. *Self-report and social desirability bias*: Questionnaire-based assessment is subject to potential overestimation of knowledge compared to actual practical skill demonstration. Participants may consciously or unconsciously inflate responses to appear more competent. The presence of research assistants during survey completion may amplify social desirability effects.

4. *Knowledge vs. performance gap*: Written knowledge assessments do not necessarily reflect actual CPR performance during emergencies. Psychomotor skills (chest compression quality, defibrillator operation), decision-making under pressure, emotional stress responses, and team coordination in real emergencies are not evaluated by written tests. Knowledge and actual performance can diverge substantially (3).

5. *Potential timing bias*: Participants who recently completed CPR training (31.2% trained within 2 years) likely demonstrate temporarily elevated scores due to fresh knowledge. Conversely, staff with knowledge decay from extended training lapse may represent particularly high-risk subgroup. Cross-sectional snapshot cannot disentangle these dynamics.

6. *Non-response bias*: While overall response rate is 90.5%, the 9.5% non-respondents characteristics are unknown. If non-responders had systematically lower knowledge or different training attitudes, results could be biased.

7. *Gender imbalance:* 73.7% of sample is female, reflecting nursing profession demographics but potentially limiting male healthcare worker perspective. Gender-specific analyses were underpowered.

8. *Sample composition*: Study included only physicians and nurses; other healthcare professionals (respiratory therapists, paramedics, biomedical technicians) were excluded due to small numbers. Results may not extend to these groups.

9. *Questionnaire validation limitations:* While CVI and Cronbach’s α are satisfactory, questionnaire was not validated against independent practical CPR skills assessments. Unknown whether written knowledge correlates strongly with actual CPR competency in this population.

10. *External validity*: Results reflect rehabilitation hospital context in Hubei Province, China. Generalizability to other Chinese regions, developing countries, or developed healthcare systems remains uncertain.

## 6 Conclusion

Healthcare professionals in tertiary rehabilitation hospitals demonstrate substantially suboptimal CPR knowledge with significant departmental and professional variations, falling substantially below international standards—creating urgent patient safety imperatives.

Professional title, department type, years of experience, and recent training independently associate with CPR competency. These results support urgent development of rehabilitation-specific, competency-based CPR training programs that address unique patient populations and clinical scenarios specific to rehabilitation contexts.

Successful implementation requires organizational commitment to addressing identified barriers, particularly workload demands and scheduling conflicts, through structural interventions rather than relying on individual motivation alone. The high training willingness (> 90%) combined with evidence-based intervention design provides realistic opportunity for meaningful competency improvement.

Future research should prioritize: (1) intervention development and RCT evaluation, (2) long-term effectiveness and sustainability assessment, (3) correlation with actual patient outcomes, and (4) multi-center validation to establish specialized emergency care competency standards for rehabilitation hospital settings and meaningfully improve patient safety in these increasingly important healthcare environments.

## Data Availability

The original contributions presented in this study are included in this article/Supplementary material, further inquiries can be directed to the corresponding author.
