# Supplementary material for: Knowledge and training willingness toward cardiopulmonary resuscitation among healthcare professionals in a tertiary rehabilitation hospital: a cross-sectional study
Source: Front Med (Lausanne). 2025 Dec 12;12:1684298. doi: 10.3389/fmed.2025.1684298 (PMC12740891; doi:10.3389/fmed.2025.1684298)
Supplement: Supplementary file 1 [file Data_Sheet_1.pdf]

Cardiopulmonary Resuscitation (CPR) Knowledge and Learning Willingness Survey Questionnaire

Tertiary Rehabilitation Hospital - Healthcare Professional Survey

---

Dear Healthcare Colleague:

Thank you for your willingness to participate in this important survey. This questionnaire aims to assess healthcare professionals' CPR knowledge levels and learning needs at our tertiary rehabilitation hospital.

Important Information:

- This survey uses anonymous response format
- All collected data will be used solely for research purposes
- Your honest and accurate responses are greatly appreciated
- Estimated completion time: 15-20 minutes

Your participation is valuable to improving emergency preparedness in our rehabilitation setting.

SECTION 1: DEMOGRAPHIC INFORMATION

1. Gender:

- □ Male
- □ Female

2. Age Group:

- □ ≤25 years
- □ 26-35 years
- □ 36-45 years
- □ ≥46 years

3. Professional Category:

- □ Physician
- □ Nurse

4. Professional Title:

- □ Junior
- □ Intermediate
- □ Associate Senior
- □ Senior

5. Educational Level:

- □ Associate degree
- □ Bachelor's degree
- □ Master's degree
- □ Doctoral degree

6. Years of Clinical Experience:

- □ ≤5 years
- □ 6-10 years
- □ 11-15 years
- □ 16-20 years
- □ ≥21 years

7. Current Department:

- □ Neurological Rehabilitation
- □ Orthopedic Rehabilitation
- □ Cardiopulmonary Rehabilitation
- □ Pediatric Rehabilitation
- □ Intensive Care Unit Rehabilitation
- □ Other (please specify): _____________

8. CPR Training in the Past Year:

- □ Yes
- □ No

9. Experience Providing CPR in the Past Year:

- □ Yes
- □ No

10. Self-Assessment of Current CPR Skill Level:

- □ Very proficient
- □ Fairly proficient
- □ Moderate
- □ Somewhat lacking
- □ Completely lacking

SECTION 2: CPR KNOWLEDGE ASSESSMENT

A. Basic Concepts and Theoretical Knowledge

1. Among the following situations, which is most likely to cause cardiac arrest in a rehabilitation patient:

- □ Excessive exercise training
- □ Psychological stress
- □ Medication adverse effects
- □ Acute exacerbation of underlying disease

2. The primary clinical sign for diagnosing cardiac arrest in a rehabilitation patient is:

- □ Loss of consciousness

- □ Rapid breathing
- □ Chest pain
- □ Decreased blood pressure

3. During rehabilitation therapy, which patient category has the highest risk of cardiac arrest:

- □ Spinal cord injury patients
- □ Stroke patients
- □ Post-myocardial infarction patients
- □ Post-orthopedic surgery patients

4. For rehabilitation patients with cardiac dysfunction, the key monitoring parameters during exercise training should include:

- □ Heart rate variability
- □ Blood pressure changes
- □ Respiratory rate
- □ Fatigue level

5. Which of the following is NOT a common risk factor for cardiac arrest in a rehabilitation hospital:

- □ Decreased exercise tolerance
- □ Autonomic nervous system dysfunction
- □ Electrolyte imbalance
- □ Acute infection

6. In pre-rehabilitation therapy assessment, which of the following indicators suggests high risk of cardiac arrest:

- □ Heart rate >120 beats/minute
- □ Systolic blood pressure >180 mmHg
- □ Oxygen saturation <90%
- □ All of the above

7. In neurological disease patients, which condition is most likely to be overlooked:

- □ Cardiac arrhythmia
- □ Autonomic dysreflexia
- □ Swallowing dysfunction
- □ Pulmonary infection

8. The primary cause of cardiac arrest in pediatric rehabilitation patients is:

- □ Respiratory failure
- □ Cardiac arrhythmia
- □ Neurogenic shock
- □ Trauma

9. When a spinal cord injury patient develops autonomic dysreflexia, the preferred body position is:

- □ Supine
- □ Head elevated 30 degrees
- □ Lateral position
- □ Sitting position

10. During exercise training for cardiopulmonary rehabilitation patients, which symptom should prompt immediate cessation of training:

- □ Mild shortness of breath
- □ Muscle soreness
- □ Anginal symptoms
- □ Increased sweating

B. Specialized Resuscitation Techniques

1. When performing CPR on a spinal cord injury patient, which measure is INCORRECT:

- □ Immediately place in supine position
- □ Remove restraining devices
- □ Maintain sitting position
- □ Remove cervical collar

2. When a patient experiences cardiac arrest during exercise training, the first action should be:

- □ Stop the exercise
- □ Call for assistance
- □ Confirm consciousness
- □ Check breathing

3. When a patient chokes during swallowing training in neurological rehabilitation, which intervention is INCORRECT:

- □ Immediately clear oral airway
- □ Adjust body position
- □ Continue training
- □ Provide oxygen

4. When performing CPR on an orthopedic rehabilitation patient wearing a support device, you should:

- □ Immediately remove all support devices
- □ Keep the support device in place
- □ Handle based on specific circumstances
- □ Wait for physician orders

5. When an accident occurs during aquatic rehabilitation therapy, the first action should be:

- □ Call for help
- □ Quickly transfer patient to dry land
- □ Provide assistance on-site
- □ Look for life-saving equipment

6. During cardiopulmonary rehabilitation training, if cardiac arrest occurs, in addition to basic life support, you also need to:

- □ Record training data
- □ Prepare defibrillation equipment
- □ Contact family members
- □ All of the above

7. When performing CPR on a polytrauma rehabilitation patient, which measure is INCORRECT:

- □ Consider cervical spine stability
- □ Be mindful of fracture sites
- □ Use standard compression depth
- □ Avoid secondary injury

8. When a critically ill rehabilitation patient on mechanical ventilation experiences cardiac arrest, you should:

- □ Immediately remove endotracheal tube
- □ Replace with manual resuscitation bag
- □ Adjust ventilator parameters
- □ Maintain original ventilation mode

9. When a patient with seizure disorder experiences cardiac arrest following seizure, the first priority should be:

- □ Restrain limbs
- □ Administer anti-seizure medication
- □ Protect airway
- □ Establish intravenous access

10. When a cardiac rehabilitation patient wearing continuous cardiac monitoring experiences cardiac arrest, you should:

- □ Immediately remove monitoring electrodes
- □ Preserve monitoring data
- □ Wait for physician arrival
- □ Ignore alarm signals

C. Special Populations and Environmental Considerations

1. Regarding cardiac arrest in pediatric rehabilitation, which statement is INCORRECT:

- □ Compression depth should be 1/3 of anterior-posterior chest diameter
- □ Use pediatric-sized AED electrode pads
- □ Compression rate is the same as in adults
- □ Compression site is the same as in adults

2. Special considerations for CPR in cerebral palsy patients do NOT include:

- □ Abnormal muscle tone
- □ Joint contracture
- □ Spinal curvature
- □ Intelligence level

3. When performing CPR on a paraplegic patient, particular attention should be paid to:

- □ Pressure ulcer status
- □ Bowel/bladder incontinence
- □ Temperature regulation
- □ All of the above

4. For orthopedic rehabilitation patients using external fixation devices, during CPR:

- □ Routinely remove the device
- □ Avoid the device as much as possible

- □ Change compression location
- □ Wait for specialist physician

5. The rehabilitation treatment room should NOT be equipped with:

- □ Automated external defibrillator (AED)
- □ Oxygen delivery equipment
- □ Emergency medications
- □ CT scanner

6. Emergency equipment for aquatic rehabilitation areas should include:

- □ Life preserver
- □ Rescue pole
- □ Stretcher
- □ All of the above

7. Special considerations for CPR in rehabilitation treatment rooms include:

- □ Remove training equipment
- □ Ensure adequate space
- □ Check floor surface safety
- □ All of the above

8. When choking/aspiration occurs during speech therapy, which intervention is INCORRECT:

- □ Immediately stop therapy
- □ Maintain original body position
- □ Use suction equipment
- □ Assess consciousness level

9. When performing CPR on an overweight rehabilitation patient, you should:

- □ Increase compression force
- □ Shorten compression duration
- □ Consider mechanical CPR device
- □ Change compression location

10. When performing CPR on a long-term bedridden rehabilitation patient, pay attention to:

- □ Osteoporosis risk
- □ Pressure ulcer status
- □ Deep vein thrombosis risk
- □ All of the above

D. Prevention and Early Warning Assessment

1. Which conditions require pre-rehabilitation cardiovascular assessment:

- □ Stroke sequelae
- □ Post-cardiac surgery
- □ Chronic obstructive pulmonary disease
- □ All of the above

2. During neurological rehabilitation, which symptoms indicate need to terminate training:

- □ Pallor (pale appearance)

- □ Speech difficulty
- □ Significant blood pressure changes
- □ All of the above

3. Pre-rehabilitation risk assessment for pediatric patients should include:

- □ Baseline disease history
- □ Exercise tolerance
- □ Family medical history
- □ All of the above

4. For cardiac rehabilitation patients, the safe heart rate range for exercise should be:

- □ Individualized by physician
- □ Based on age-matched standards
- □ Calculated using fixed formula
- □ Determined by patient preference

5. Which patients are NOT suitable for aquatic rehabilitation therapy:

- □ Patients with open wounds
- □ Patients with fever
- □ Patients with severe arrhythmia
- □ All of the above

6. Prevention measures for high-risk patients during rehabilitation training should include:

- □ Develop emergency response plan
- □ Prepare monitoring equipment
- □ Clarify management responsibilities
- □ All of the above

7. The recommended frequency for rehabilitation treatment room emergency drills should be:

- □ Monthly
- □ Quarterly
- □ Semi-annually
- □ Annually

8. Which item is NOT essential monitoring equipment for rehabilitation treatment rooms:

- □ Blood pressure monitor
- □ Cardiac monitor
- □ Pulse oximeter
- □ Mechanical ventilator

9. During high-intensity exercise training, primary monitoring parameters should include:

- □ Heart rate changes
- □ Respiratory rate
- □ Blood pressure fluctuations
- □ All of the above

10. When rehabilitation patients present with the following conditions, training should be adjusted or suspended:

- □ Increased fatigue

- □ Decreased activity tolerance
- □ Changes in sleep quality
- □ All of the above

E. Team Coordination and Emergency Response

1. When requesting assistance after discovering cardiac arrest, the information to provide should include:

- □ Specific location
- □ Patient condition
- □ Measures already taken
- □ All of the above

2. Healthcare personnel who need to master basic life support skills include:

- □ Physicians
- □ Nurses
- □ Rehabilitation therapists
- □ All of the above

3. During CPR, the most important aspect of team cooperation is:

- □ Clear role assignment
- □ Effective communication
- □ Timely coordination
- □ All of the above

4. Who should be responsible for managing emergency supplies in rehabilitation treatment rooms:

- □ Physician
- □ Nurse
- □ Rehabilitation therapist
- □ Designated personnel

5. The record keeper should prioritize documenting:

- □ Time of discovery
- □ Interventions and medications
- □ Patient response
- □ All of the above

6. Post-CPR team debriefing should NOT include:

- □ Resuscitation procedures
- □ Team coordination effectiveness
- □ Patient outcome
- □ Medical insurance costs

7. Management measures for high-risk patient rehabilitation training should include:

- □ Clarify responsible personnel
- □ Develop emergency response plan
- □ Prepare emergency equipment
- □ All of the above

8. The most important element when conducting CPR training is:

- □ Theoretical knowledge
- □ Operational skills
- □ Team cooperation
- □ All of the above

9. Emergency drills in rehabilitation treatment rooms should include:

- □ Role assignment
- □ Process rehearsal
- □ Equipment operation
- □ All of the above

10. The most critical factor for improving emergency response capability in rehabilitation treatment rooms is:

- □ Improve institutional policies
- □ Strengthen training programs
- □ Equip with necessary devices
- □ All of the above

SECTION 3: LEARNING WILLINGNESS AND TRAINING NEEDS SURVEY

A. Cognitive Attitudes Assessment

Instructions: Please indicate your level of agreement with each statement by checking (√) the corresponding number:

- 1 = Strongly disagree
- 2 = Somewhat disagree
- 3 = Neutral/Uncertain
- 4 = Somewhat agree
- 5 = Strongly agree

1. I understand the necessity of CPR training for rehabilitation hospitals

- 1 □ 2 □ 3 □ 4 □ 5 □

2. I believe improving CPR ability is important for patient safety

- 1 □ 2 □ 3 □ 4 □ 5 □

3. I am interested in learning CPR-related knowledge and skills

- 1 □ 2 □ 3 □ 4 □ 5 □

4. I am willing to invest time to improve my CPR ability

- 1 □ 2 □ 3 □ 4 □ 5 □

5. I believe periodic CPR training and assessment is necessary

- 1 □ 2 □ 3 □ 4 □ 5 □

B. Training Needs Analysis

1. Which areas would you most like to improve? (Multiple selections allowed)

- □ Recognition of cardiac arrest
- □ Basic life support skills
- □ Team coordination
- □ AED operation
- □ CPR for special populations
- □ Emergency equipment use
- □ Emergency protocol development
- □ Other (please specify): ______________

2. Which training content do you believe needs most emphasis? (Multiple selections allowed)

- □ Recognition of critical conditions in rehabilitation patients
- □ Prevention and management of complications specific to rehabilitation
- □ CPR techniques for special populations
- □ Team coordination procedures
- □ Emergency equipment operation
- □ Emergency protocol drills
- □ Case discussion and analysis
- □ Other (please specify): ______________

3. What training format do you prefer? (Multiple selections allowed)

- □ Expert lectures
- □ Video teaching
- □ Simulation training
- □ Case-based discussion
- □ Practical drills
- □ Performance feedback/testing
- □ Online learning
- □ Other (please specify): ______________

4. What is your preferred duration for a single training session:

- □ Less than 1 hour
- □ 1-2 hours
- □ 2-4 hours
- □ More than 4 hours

5. What training frequency would you prefer:

- □ Monthly
- □ Quarterly
- □ Semi-annually
- □ Annually

6. When would it be most convenient for you to participate in training:

- □ Weekday mornings
- □ Weekday afternoons
- □ Weekday evenings
- □ Weekends

7. What factors most affect your ability to participate in training? (Multiple selections allowed)

- □ Heavy workload
- □ Scheduling conflicts with clinical duties
- □ Limited training method variety
- □ Content lacks relevance to my work
- □ Limited hands-on practice opportunities
- □ Lack of assessment incentives
- □ Personal motivation issues
- □ Other (please specify): ______________

C. Open-Ended Questions

1. What do you believe are the main gaps in emergency response capacity in your current department?

---

2. What suggestions do you have for improving CPR ability among healthcare professionals in rehabilitation hospitals?

---

3. What special scenarios do you believe require focused training programs?

---

4. What suggestions do you have for improving emergency response protocols in rehabilitation treatment rooms?

SURVEY COMPLETION INFORMATION

Date of Completion: _____ Year _____ Month _____ Day

Identification Number (optional): ____________________

THANK YOU FOR YOUR PARTICIPATION

Thank you for taking the time to complete this survey. Your candid responses are invaluable for enhancing emergency preparedness and improving patient safety in our rehabilitation hospital.

If you have any questions or suggestions regarding this research, please contact:

Principal Investigator Contact Information:

- Name: ____________________
- Department: ____________________
- Phone: ____________________
- Email: ____________________

RESEARCH ETHICS NOTICE

This study has been approved by the Ethics Committee of Taihe Hospital (Approval No. WZ-2024-045) and complies with the Declaration of Helsinki. Your participation is entirely voluntary, and you may withdraw at any time without penalty or loss of benefits. Your responses will be kept confidential and anonymous.
